# Treatments, Perceived Stigma, and Employment Outcomes among Substance Abusers in China

**DOI:** 10.3390/healthcare10010130

**Published:** 2022-01-09

**Authors:** Li Han, Cindy Xinshan Jia

**Affiliations:** Department of Social Work, College of Public Management, South China Agricultural University, Guangzhou 510640, China; lynn_han1216@scau.edu.cn

**Keywords:** substance abuse treatment, employment, perceived stigma

## Abstract

Employment is a vital component of a substance abuser’s recovery, but little is known about how stigma affects employment for substance abusers receiving treatment. The current study investigates the effects of stigma and treatment on employment in the Chinese context. Using a sample of substance abusers (*N* = 3.978), multiple logistics regressions with moderation effects were employed. The findings show that treatments positively reduce confirmative experiences of anticipated stigma, and promote employment only when respondents do not perceive stigma. The findings highlight the impact of perceived stigma on limiting substance abusers’ chances of being employed, implying that eliminating stigma is the foundation for recovery. Possible strategies that can be explored for reducing stigma are discussed.

## 1. Introduction

Employment promotes a substance abuser’s recovery, and serves as one of the most important outcome criteria for substance abuse treatment [1]. Substance abuse raises the likelihood of unemployment [2], whereas unemployment, in turn, has a negative impact on the health and social well-being of substance users [3]. Gaps in employment and employment history, a possible criminal record, and the stigma connected with substance use, all make substance abusers less likely to keep their current jobs, and more likely to have trouble finding new work in the future [2]. To improve the employment status among substance abusers, numerous treatments have been offered [2,4,5]; However, the effect size tended to be modest [4], and relapses from treatments looked to be significant [6,7]. A relevant body of literature has identified several attributes explaining such failures, such as beliefs about one’s personal coping ability [8], failures in abstinence [9], an inability to afford the treatment, and a lack of relevant information [10], as well as the negative impacts caused by stigma [8]. Among these factors, there has been little attention to how stigma has influenced the effects of treatment on a substance abuser’s employment.

### 1.1. Stigma of Substance Abuse

Stigma appears to be one of the greatest barriers for substance abusers considering recovery [11]. Stigma refers to a “mark” or an attribute that defines an individual with stereotypes that tend to lead to discriminatory behavior against that individual [12,13,14]. Substance abusers frequently face public disapproval due to pre-existing stereotypes, such as being dangerous and irresponsible [15,16]. Substance abusers who are aware of such stereotypes may engage in personal devaluation, and internalize these stigma [17]. As a result, stigma damages several aspects of a substance abuser’s life, limits their opportunities for recovery, and leads to poor health outcomes [15,17].

Perceived stigma serves as a key component of the self-stigma process through a mechanism of self-fulfilling prophecy [14,18]. The self-fulfilling prophecy proposes that, with an awareness of stereotypes, individuals may anticipate public stigma, such as assuming the status of being in an inferior group or expecting discrimination, and they subsequently internalize the stigma, that is, a self-stigmatization process, when their experiences confirm their anticipation/expectation (i.e., an assumption-confirming process) [19,20]. As a conceptual form of stigma, perceived stigma refers to an individual’s beliefs and perceptions regarding the prevalence of stigmatized attitudes and behaviors others hold toward them [21]. Perceived stigma significantly contributes to internalized stigma (i.e., self-stigma) [17]. With the experience of social rejection (i.e., enacted stigma), individuals develop an awareness of possible stigma [21], such as discrimination in treatments, and being denied a job [22,23]. Then, perceived stigma is formed through the above-mentioned assumption/expectation–confirmation process of self-fulling prophecy. As a result, self-stigma develops as individuals repeatedly encounter and confirm their anticipated stigma (i.e., confirmative experiences of perceived stigma) [17,24,25].

Self-stigma leads to various impairments among substance abusers. For example, it harms individuals’ self-functions, creates a personal sense of guilt and feelings of hopelessness; promotes self-devaluation, anxiety, and depression; and encourages withdrawal from social support, rejection of help, and avoidance of and withdrawal from treatment, all of which limit substance abusers’ chances for recovery [15,17,26,27,28]. Furthermore, the stigma process influences the employment prospects of substance abusers. As they ascribe stereotypes and beliefs about their own inferiority to themselves, and anticipate discrimination and rejection, these self-stigmatized substance abusers tend to have limited social networks, expect to be rejected in social situations, fail to secure employment, and experience relapses from treatment [10,16,29,30], which increases their self-stigma even further [21].

Perceived stigma possibly moderates the impact of treatment on employment [31]. Individuals who are aware of stigma may not necessarily develop self-stigma, and some will resort to ignorant reactions to cope with enacted stigma [32]. According to the expectation–confirmation process of self-stigma, whether enacted stigma is confirmed or not depends on the magnitude of the perceived stigma. For example, one study explored the self-stigma of drug use and HIV status, and found that individuals in a condition of higher self-stigma benefited less from drug treatment [33]. Other factors promoting the development of self-stigma include psychological symptoms [22], perceived primal threat [34], substance usage [35], the long period of one’s substance abuse [36], and negative self-concepts of dysfunction [37]. Given the importance of employment in substance abusers’ recovery, it is worthwhile to research how the perceived stigma influences the effects of treatment on a substance abuser’s employment.

### 1.2. Substance Abuse and Stigma in China

China has a growing population of registered substance abusers (i.e., individuals who have been medically diagnosed with an addictive disorder, and registered in an administrative system for substance abusers), from 1.16 million in 2005 to 2.1 million in 2012 [38]. Regarding substance abuse treatment, there are compulsory and voluntary types available, depending on an individual’s substance abuse diagnosis and criminal history. For substance abusers with a history of arrest or a criminal record, compulsory treatment programs are required by the country’s law enforcement agencies [39]. For those who have severe substance abuse, a Compulsory Institutional Drug Treatment program (Institutional Treatment) is required. It generally includes physical recovery, psychological education, and abstinence training for severe substance abusers, and usually lasts for two years. For minor substance users and individuals who completed the institutional treatment, the Community-based Drug Treatment and Rehabilitation program (Community Treatment) is required. The community treatment usually lasts for three years, with an individualized plan targeting community reentry, includes psychological education and counseling for health, family relationships, and employment. Herein, in community treatment, local administrators and social work agencies usually provide two optional voluntary treatments. One is community reentry (Reentry Treatment), which usually includes abstinence supervision, psychological education, and counseling. The other is employment treatment (Employment Treatment), which usually includes employment training in seeking job opportunities, and interview and job skills, depending on an assessment and the needs of the substance abuser. Nevertheless, most of the literature on treatment effects among Chinese substance abusers has focused on the effects of pharmaceutical treatment, such as Methadone Maintenance Treatment [40,41,42]. The impact of non-pharmaceutical programs has barely been investigated [43,44].

Chinese substance abusers, like substance abusers in other countries, face stigma and discrimination [40,45,46,47]. Chinese people, who live in a collective cultural society, develop self-construal with an emphasis on social relationships, and social devaluation is thus particularly harmful to their well-being [28,48]. China’s policy on registering substance abusers exacerbates the self-stigma process, as such a registration system is seen as a lifelong “stamp” [49,50], which influences substance abusers’ experience of stigma at multiple levels, including family, friends, professional groups, their communities, and society [51,52,53]. As a result, such stigma is associated with greater self-stigma and self-labeling [54,55], dropping out of treatment programs [56], and a higher risk of unemployment [50]. However, how such stigma influences treatment among substance abusers remains largely unknown in the Chinese context.

### 1.3. The Current Study

**Figure 1:**
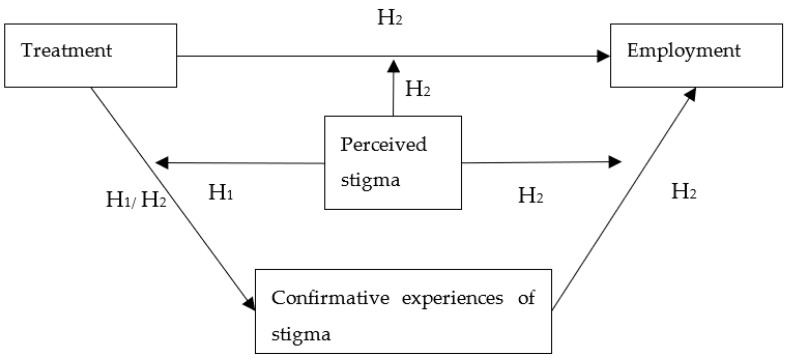
Hypothetical framework.

The current study explores how perceived stigma moderates the relationship between treatment and employment among Chinese substance abusers. Specifically, according to the self-fulfilling process, this study investigates how perceived stigma, as a moderator, influences the relationship between treatment and the confirmative experience of anticipated stigma (i.e., the expectation–confirmation process of self-stigma), as well as between treatment and employment status (see Figure 1). It develops two hypotheses:

**Hypothesis** **1** **(H1).** *Perceived stigma moderates the positive effects of treatment in reducing the confirmative stigma experience in substance abusers’ process of finding employment, such that treatment reduces the confirmative experiences of stigma only when there is no perceived stigma*.

**Hypothesis** **2a** **(H2a).** *Perceived stigma moderates the positive effects of treatment on substance abusers’ employment outcomes, such that treatment promotes employment only when there is no perceived stigma*.

**Hypothesis** **2b** **(H2b).** *Perceived stigma moderates the effects of confirmative experiences of stigma on substance abusers’ employment outcomes, such that the absence of confirmative experiences of stigma promote employment only when there is no perceived stigma*.

**Hypothesis** **2c** **(H2c).** *The absence of confirmative experiences of stigma mediates between treatment and employment*.

## 2. Materials and Methods

### 2.1. Data and Sample

The current study obtained ethical approval from the Institutional Review Board (IRB) of the Anthropology Department at Sun Yat-sen University, China. We employed a cross-sectional design. A two-stage cluster sampling frame was applied: the first and second sampling units were “city” and “street”. Individuals who received any substance abuse treatment were referred by two province-level agencies in Guangdong Province. Eight cities in Guangdong were randomly selected, fifteen street-level units were randomly selected per city, and ten to fifty individuals were randomly selected from every street unit. Using voluntary online questionnaires with informed consent, a total of 6128 responses were collected, and 3217 were maintained after a screening for duplicates, and a response time check (i.e., observations with response time between M ± 2SD retained).

### 2.2. Measurement

#### 2.2.1. Dependent Variables

*No confirmative stigma experiences.* Confirmative stigma experiences were measured using a single question that asked respondents about their experiences with stigma in the job-finding process. The responses were originally rated on the following scale of degree of stigma severity: “not severe at all” = 0, “not severe” = 1, “severe” = 2, and “extremely severe” = 3. As the rating scale was bi-dimensional in form, dummy coding was adopted to clarify the presence of confirmative experiences of stigma. Further, as having treatment was expected to positively promote employment status, in order to clarify moderation directions, “not severe at all” and “not severe” were coded as 1, and “severe” and “extremely severe” were coded as 0.

*Employment status.* Respondents’ employment status was measured by a single question asking about the name of their current job. A score of 1 was given if the respondent reported a concrete job, and 0 was given if the respondent reported being unemployed, in the process of looking for a job, or staying at home. A blank report was coded as a missing value.

#### 2.2.2. Independent Variables

*Treatments.* The current survey asked about the perceived effectiveness of the four types of treatment: the Community-based Drug Treatment and Rehabilitation program (Community Treatment); the Compulsory Institutional Drug Treatment program (Institutional Treatment); a program with administrative assistance in community reentry and integration (Reentry Treatment); and a program specifically focused on employment training (Employment Treatment). The effectiveness of these programs was dummy coded such that a self-report of effect was coded as 1, whereas a report of no effect was coded as 0.

*No perceived stigma*. As mentioned above, stigma encompasses various social levels [52]. The survey employed the most influential levels of family, society, and official propaganda, and asked about the degree to which respondents perceive stigmatizing attitudes and actions from these three sources. It was originally rated on a bi-dimensional 4-point scale from “not at all” = 1 to “very much” = 4. The four levels yielded a solid internal consistency (i.e., Cronbach’s α = 0.85), and the composed score was computed. Notably, as non-stigmatization is intuitive in explaining the positive effects of treatment on dependent variables, following the common dummy coding of perceived stigma [57,58], negative responses (i.e., there is no perceived stigma, i.e., “not at all” or “not much”) were coded as 1, whereas positive responses (i.e., perceived stigma, i.e., “somewhat” or “very much”) were coded as 0.

#### 2.2.3. Controlled Covariates

*Psychological symptoms*. As psychological symptoms are associated with stigma among individuals with substance abuse [59], anxiety, depression, and somatization were measured by the Brief Symptom Inventory 18 (BSI-18) [60]. Respondents rated on a 5-point frequency scale their experiences of psychological symptoms in the recent week, from “not at all” = 0 to “very frequently” = 4. Composed scores for three subscales were achieved with good internal consistency (i.e., Cronbach’s αs of 0.91, 0.91, and 0.92 on anxiety, depression, and somatization, respectively).

*Duration of abstinence*. The duration of abstinence was controlled, as it influences stigma [61]. The participants were asked about their estimated days of abstinence. As the participants on average have a three-month abstinence period (SD = 1.03), days were transferred into months for analysis.

*Type of substance*. The type of substance that is abused influences the stigma process [33]. According to the substance usage representativeness in the current sample, the substances were classified as one of three types: heroin, methamphetamine, and other. Other substance includes opium, cocaine, cannabis, morphine, ketamine, ecstasy, and other psychoactive substances. The categories were dummy coded so that a history of usage = 1, and no history of usage = 0.

*Demographic characteristics.* Respondents were asked about demographic factors, such as gender, education, age, income, and marital and fertility status. Gender was coded as “male” = 1 and “female” = 0. Education was coded into years of education as “Primary school or below” = 9, “High school/vocational school” = 12, “College degree/Diploma” = 15, “Undergraduate education/Bachelor” = 16, and “Postgraduate education/Master or above” = 19. Age was coded into years of age. Income was coded according to respondents’ personal monthly income. Furthermore, as there was a limited number of respondents who were divorced or had a marital status other than married (1.18%), marital status was coded as “married” = 1 and “other” = 0. The respondents were also asked about their fertility status: “have at least one child” was coded as 1, and “do not have any children” was coded as 0.

### 2.3. Data Analysis

Logistic regressions were performed to test the hypotheses with Stata 15.0. Regarding the tests of the moderation effects in H1, H2a, and H2b, three steps were performed for the two dependent variables (i.e., no confirmative experience of stigma and employment status) to test the hypotheses: (a) the effects of covariates and demographic variables; (b) the effects by adding the main effect terms (i.e., treatments and no perceived stigma for H1; treatments, no perceived stigma, and no confirmative experience of stigma for H2); and (c) the effects by adding the interaction terms. Regarding the complexity of interpreting interaction terms in logistic regression, the moderation effect of the interaction term of *x*1 and *x*2 was computed following Haile [62]: $OR_{x1x2}=e^{\beta_{x1}}e^{\beta_{x2}}e^{\beta_{x1x2}}$. Regarding the test of the mediation effect in H2c, the Karlson–Holm–Breen (KHB) method was used to examine the direct and indirect effects [63,64].

## 3. Results

Regarding the missing data, blank observations were deleted. The percentage of missing values was low (i.e., less than 0.01%) for the variables included in the analysis, and the missing data was auto-deleted in the analyses.

Descriptive statistics were presented in Table 1. Regarding demographic characteristics, the sample of substance abusers was predominantly male (i.e., 91.99%), with an average age of 38 (SD = 0.15). They generally had a secondary school education (i.e., 8.55 years of education) and an average level of monthly income (i.e., M = 4.34, SD = 1.47). Over half of the sample was married (i.e., 56.40%) and had at least one child (i.e., 65.02%). Regarding the substance type, use of heroin accounted for one-third, use of methamphetamine accounted for half, and the use of other types of substances accounted for one-sixth of the sample. Having a perception and experience of stigma was prevalent among respondents (i.e., 46.10% and 56.40%, respectively). The majority of respondents had engaged in one of the four types of treatment (i.e., 89.68~92.94%).

Regarding H1, Table 2 presents three models showing the effects of controlled covariates, and independent and moderation variables on no confirmative experiences of stigma. In general, adding the main effect terms (i.e., Model 2 in Table 2) and interaction terms (i.e., Model 3 in Table 2) significantly increased the model fit (χ^2^ = 676.83, *p* < 0.00; χ^2^ = 17.19, *p* < 0.00), indicating that treatments and no perceived stigma, as well as their interaction term, significantly improved the explanation of no confirmative experiences of stigma. Regarding the main effect, Model 2 showed that individuals without perceived stigma have 6.2 times higher odds of not confirmatively experiencing stigma when finding a job. Model 3 showed that when considering the moderation effect, individuals without perceived stigma have 2.72 times higher odds of not confirmatively experiencing stigma when finding a job. Those who were not engaged in the employment treatment had 1.42 times higher odds of experiencing confirmative stigma when finding a job (OR = 0.70, *p* < 0.05). Also, the odds ratio of the interaction term between the employment treatment and no perceived stigma was computed, and appeared as 3.53, *p* < 0.00, which indicated that compared to others, individuals without perceived stigma who had also engaged in employment treatment had 3.53 times higher odds of not experiencing confirmative stigma when finding a job. However, it did not reveal any other significant treatment effect. Thus, H1 was partially supported.

Regarding H2, Table 3 presents three models showing the effects of controlled covariates, and independent and moderation variables on employed status. In general, adding the main effect terms (i.e., Model 2 in Table 3) significantly increased the model fit (χ^2^ = 39.48, *p* < 0.00), indicating that treatment and perceived stigma significantly improved the explanation of employed status. Regarding the main effect, Model 2 showed that no confirmative experiences of stigma and no perceived stigma were slightly negative, but significantly predicted employed status with OR = 0.81 *p* < 0.05 and OR = 0.99 *p* < 0.00, respectively. Individuals involved in the community and employment treatments promoted employed status by 1.65 and 2.07 times higher odds, respectively (OR = 1.65, *p* < 0.00 and OR = 2.07, *p* < 0.00). Model 3 showed that when taking the moderation effect into consideration, individuals who engaged in employment treatment had 2.01 times higher odds of becoming employed. Also, the odds ratio of the interaction term between the community treatment and no perceived stigma was computed, and appeared as 2.39, *p* < 0.05, which indicated that compared to other individuals, those without perceived stigma who were also engaged in community treatment had 2.38 times higher odds of being employed. However, it did not reveal any other significant treatment effect. Thus, H2a and H2b were partially supported. Furthermore, KHB tests with bootstrap (50 replications) showed a significant direct effect of employment treatment predicting employed status that, engaging in employment treatment significantly increased the log odds of being employed by 0.70 with *p* < 0.05. There was no significant direct effect revealed regarding other treatments. Also, no significant indirect effect of not experiencing confirmative stigma mediating between treatments and employed status was found. These results were consistent with logistic model results. Thus, H2c was not supported.

Regarding the covariates, Model 3 in Table 2 reveals the negative effects of depression (OR = 0.56, *p* < 0.00) on no confirmative experiences of stigma, such that one unit of increase in depression results in 1.79 times higher odds of having confirmative experiences of stigma. Also, being female and having married status appeared to have 1.72 and 1.22 times higher odds, respectively, of not confirmatively experiencing stigma. Model 3 in Table 3 revealed that heroin users and younger individuals had 1.44 and 1.02 times higher odds, respectively, of being employed.

## 4. Discussion

Employment is one of the most vital protective factors in a substance abuser’s recovery. The current study is one of the first to examine the treatment effects on employment with the moderation of perceived stigma among Chinese substance abusers. The findings suggest that perceived stigma strongly accelerates the process of self-stigma, such that individuals with perceived stigma were more likely to confirm their anticipated stigma. Also, the positive effects of treatment were observed only among those who did not perceive stigma: the employment and community treatments positively reduced the confirmative experiences of stigma and promoted employment, respectively, but only for those who did not have perceived stigma. The findings have several research and practical implications.

First, the study elaborates on the mechanism of the expectation-confirming process of the self-fulling prophecy in forming self-stigma. It does so by highlighting the moderation effect of stigma perceptions. As noted, enacted stigma does not always lead to the confirmation of stigma experience, and individuals employed other reactions as coping strategies, such as anger and ignorant reactions [32]. The current study revealed that perceived stigma matters, such that only those who were concerned about or believed that public stigma exists were significantly less likely to benefit from their treatments in promoting employment. This finding echoes Schomerus’s self-stigma model of substance use by highlighting perceived stigma [16]. Schomerus’s model showed that individuals agree and internalize stereotypes only under the condition of stereotype awareness, which is a type of stigma perception [65]. Other studies, such as that by Chi and colleagues [24], also suggested a dynamic self-stigma circle that includes enacted stigma, perceived stigma, and enacted stigma, which aligns with the current findings regarding the impact of perceived stigma on confirmative experiences of stigma. However, the mediation effect of the confirmatory experience of stigma was not supported in the current model, implying partial support of the expectation–confirmation process in developing self-stigma. As other studies suggested empirical evidence for such a process [17,21], future studies could explore the causal factors in the self-stigma process, such as the mechanisms among stigma perceptions, confirmations, and outcomes, as well as other factors, such as experiential avoidance [66].

Second, regarding the types of treatment available to Chinese substance abusers, the current study implies that there is a positive, thought slight, direct effect of employment and community treatment in decelerating the self-stigma process, and improving employment, net of other treatments, perceived stigma, and confirmatory experiences of stigma, which is consistent with previous findings in other cultural contexts [5]. Nevertheless, it is vital to emphasize that such beneficial effects of treatment may not apply to other parts of mainland China. As noted above, the research site for the current study was Guangdong province, which is one of the first provinces to offer social work substance services in mainland China. Involving professionals such as social workers, counselors, and therapists is still new and under-developed for much of the rest of the country [38]. In addition, regarding the critics of the compulsory nature of substance abuse treatments [38], the current study did not reveal any negative evidence, implying potential benefits of such treatments. Further empirical evidence regarding the substance abuse treatment effects in mainland China is needed [44].

Third, the effects of symptoms of depression and substance abuse status on stigma are noteworthy. The current findings demonstrated a strong association between depression and confirmative experiences of stigma, which is consistent with prior findings on the stigma of HIV and alcohol use [24,67]. Moreover, the types and duration of substance use may influence the stigmatization process. In comparison to heroin users and others (i.e., opium, cocaine, cannabis, morphine, ketamine, ecstasy, and other psychoactive substances), methamphetamine users have a slightly lower risk of self-stigma (see Table 2), which is partially consistent with Brown’s [65] finding that self-stigma was greater among heroin users than marijuana users. However, the findings also revealed that heroin users had a slightly higher chance of being employed than users in other categories (see Table 3), which contradicts prior findings [68]. One possible explanation is that the current sample includes individuals who have previously taken drugs, and heroin is a kind of “traditional” drug substance. The previous heroin users are significantly older (*M* = 43.41) than other users (*M* = 35.33), with a *t*-value of 29.94, *p* < 0.00. Individuals in their forties and fifties who maintain their abstinence are often under more pressure to work in order to support their families. Nonetheless, it is worth mentioning that the current study did not include users of other substances such as tobacco, alcohol, or other such substances, which require further investigation.

The current study contributes to the body of literature on substance abuse treatment in mainland China by shedding light on the effectiveness of substance abuse treatment in improving employment. Treatment for substance abusers, such as methadone maintenance treatment and social work services, is still at an early stage of development [38]. The current study primarily suggests that compared to institutional treatment and rehabilitation, community-based programs, which typically include social work services targeting employment, family relationships, medical status, and other areas, significantly improved clients’ recovery process in terms of reducing stigma, and improving employment. Nevertheless, an assessment of the effects of non-pharmaceutical treatment among substance abusers in China is still rare [44]. Given the insignificant finding that confirmatory experiences of stigma failed to mediate between treatments and employment, further empirical investigation should consider non-pharmaceutical treatment effects in individual rehabilitation mechanisms in both mental and social aspects, such as reducing self-stigma, and promoting family relationship and employment. Also, given the significant moderation effect of perceived stigma, evidence-based interventions with assessments of the effects of reducing stigma and promoting employment [69,70], such as acceptance and commitment therapy [11], and psychological education with cognitive behavioral therapy [71], should be considered in the future.

Several limitations of the current study should be noted. First, it employed a self-report questionnaire survey method, which could lead to a bias in the responses, such as the tendency toward social desirability. Second, the study was cross-sectional, and the treatment effects need further investigation through a longitudinal or experimental design, such as randomized control trials. Third, the measurements need improvement in future studies, such as using the perceived stigma scale [21], and including tobacco and alcohol as types of substances that are also abused. Finally, cross-sectional comparisons, especially including areas with less development in helpful non-pharmaceutical professional services, such as social work and counseling, are worthy of future research to examine the treatment effects nationally among substance abusers.

## 5. Conclusions

As employment promotes substance abusers’ recovery, the current study aimed to examine how perceived stigma affects the positive effects of treatment on employment. Employing a sample of substance abusers (*N* = 3.978) in China’s Guangdong province, the current study highlighted the moderation role of perceived stigma, finding that the positive effects of treatment in reducing the self-stigma process in the job search process, and in promoting employment, occurs only among those who did not perceive stigma. This conclusion implies that implementing community and employment treatments for substance abusers is beneficial for their recovery. Also, reducing stigma should be regarded as the foundation for such substance abuse treatment. Interventions reducing stigma perceptions should be implemented throughout different types of substance abuse treatment processes in institutes or communities. Also, public administration organizations should consider using appropriate anti-substance abuse messaging to reduce the exaggerated public stigma.

## Figures and Tables

**Table 1:** Sample descriptions.

	Female	Male	Total
*N*	339 (8.01%)	3893 (91.99%)	4232 (100.00%)
*N* of employed	239 (70.50%)	2974 (76.39%)	3217 (76.02%)
*N* having confirmative experiences of stigma	163 (48.08%)	2216 (56.92%)	2387 (56.40%)
*N* participating in treatment			
Community treatment	315 (92.92%)	3609 (92.70%)	3933 (92.94%)
Institutional treatment	314 (92.63%)	3601 (92.50%)	3924 (92.72%)
Reentry treatment	304 (89.68%)	3571 (91.73%)	3884 (91.78%)
Employment treatment	308 (90.86%)	3505 (90.03%)	3821 (90.29%)
*N* not having perceived stigma	154 (45.43%)	1794 (46.08%)	1951 (46.10%)
*M*s of psychological symptoms			
Anxiety	1.31 (0.03)	1.28 (0.01)	1.27 (0.01)
Depression	1.31 (0.03)	1.29 (0.01)	1.30 (0.01)
Somatization	1.26 (0.03)	1.26 (0.01)	1.26 (0.01)
*M* of abstinence duration (month)	3.28 (0.06)	3.33 (0.02)	3.32 (0.02)
*M* of personal monthly income (1000 CNY)	3.94 (0.09)	4.37 (0.02)	4.34 (1.47)
*M* of years of education	8.81 (0.17)	8.53 (0.05)	8.55 (0.05)
*M* of years of age	33.85 (0.47)	38.36 (0.15)	38.00 (0.15)
*N* of married	159 (46.90%)	2224 (57.13%)	2387 (56.40%)
*N* having at least one child	190 (56.05%)	2558 (65.71%)	2752 (65.02%)

Note. Percentages of the category total and standardized errors are in parentheses. CNY = Chinese Yuan (1 CNY ≈ 0.16 USD). *N* = number, and *M* = mean value.

**Table 2:** Logistic regressions with robust standard error for perceived stigma moderates treatment, predicting no confirmative experiences of stigma (*N* = 3.978).

	Model 1		Model 2		Model 3	
Predictors	OR	95% C.I.	OR	95% C.I.	OR	95% C.I.
Psychological symptoms						
Anxiety	0.82	0.59~1.12	0.86	0.61~1.21	0.87	0.62~1.22
Depression	0.46 ***	0.34~0.62	0.57 ***	0.42~0.79	0.56 ***	0.41~0.77
Somatization	1.09	0.84~1.43	1.18	0.88~1.57	1.18	0.88~1.57
Duration of abstinence (month)	1.04	0.97~1.11	1.00	0.93~1.07	1.00	0.94~1.08
Type of substance (Base = *no usage*)						
Heroin	1.10	0.92~1.31	1.16	0.95~1.41	1.15	0.94~1.39
Methamphetamines	0.84 *	0.72~0.99	0.94	0.79~1.13	0.95	0.79~1.13
Other	1.02	0.84~1.24	1.05	0.85~1.29	1.04	0.84~1.28
Demographics						
Male (Base = *female*)	0.63 ***	0.49~0.80	0.57 ***	0.44~0.75	0.58 ***	0.45~0.76
Have at least one child (Base = *none*)	0.88	0.73~1.06	0.92	0.75~1.14	0.92	0.75~1.14
Married (Base = *others*)	1.13	0.95~1.36	1.21	0.99~1.47	1.22 *	1.00~1.49
Personal monthly income (CNY)	1.00 ***	1.00~1.00	1.00 ***	1.00~1.00	1.00 ***	1.00~1.00
Years of education	1.01	0.98~1.03	1.01	0.98~1.03	1.01	0.98~1.04
Years of age	1.00	0.99~1.01	0.99	0.98~1.00	0.99	0.98~1.00
Treatments (Base = *not* *participating in* *one of the treatments below*)						
Community treatment			1.04	0.75~1.45	1.10	0.71~1.72
Institutional treatment			0.88	0.64~1.23	0.85	0.56~1.29
Reentry treatment			1.05	0.74~1.47	0.88	0.58~1.35
Employment treatment			1.00	0.74~1.36	0.70 *	1.00~2.03
No perceived stigma (Base = *having perceived stigma*)			6.20 ***	5.36~7.16	2.27 **	0.49~1.00
Moderation terms						
Community treatment × No perceived stigma					0.82	0.42~1.60
Institutional treatment × No perceived stigma					1.17	0.62~2.20
Reentry treatment × No perceived stigma					1.42	0.76~2.68
Employment treatment × No perceived stigma					2.22 ***	1.29~3.81
Goodness-of-fit	$\chi^{2}$		$\chi^{2}$		$\chi^{2}$	
Wald test (*df*)	181.29 *** (13)		766.95 *** (18)		782.24 *** (22)	
Likelihood ratio (*df*)			676.83 *** (5)		17.19 *** (4)	

Note. *** *p <* 0.00; ** *p <* 0.01; * *p <* 0.05. C.I. = the confidence interval. Likelihood ratio tests were conducted for logistic regression without robust standard error.

**Table 3:** Logistic regressions with robust standard error for no perceived stigma moderating treatment and no confirmative experience of stigma predicting employment status (*N* = 3.978).

	Model 1		Model 2		Model 3	
Predictors	OR	95% C.I.	OR	95% C.I.	OR	95% C.I.
Psychological symptoms						
Anxiety	0.90	0.64~1.28	0.94	0.66~1.33	0.94	0.66~1.34
Depression	0.91	0.69~1.19	0.88	0.66~1.16	0.88	0.67~1.16
Somatization	0.89	0.65~1.21	0.93	0.68~1.27	0.92	0.68~1.26
Duration of abstinence (month)	0.96	0.88~1.04	0.95	0.88~1.04	0.95	0.88~1.03
Type of substance (Base = *no usage*)						
Heroin	1.40 ***	1.11~1.78	1.44 ***	1.13~1.84	1.44 ***	1.13~1.84
Methamphetamines	1.20	0.96~1.49	1.18	0.95~1.48	1.18	0.95~1.48
Other	0.96	0.75~1.24	0.98	0.75~1.27	0.98	0.76~1.27
Demographics						
Male (Base = *female*)	1.09	0.81~1.48	1.07	0.79~1.46	1.07	0.79~1.46
Have at least one child (Base = *none*)	1.01	0.79~1.30	1.04	0.81~1.34	1.04	0.81~1.33
Married (Base = *others*)	1.24	0.98~1.57	1.23	0.97~1.57	1.24	0.98~1.57
Personal monthly income (CNY)	1.00 ***	1.00~1.00	1.00 ***	1.00~1.00	1.00 ***	1.00~1.00
Years of education	0.97	0.94~1.01	0.97	0.94~1.01	0.97	0.94~1.01
Years of age	0.98 ***	0.97~0.99	0.98 ***	0.97~0.99	0.98 ***	0.97~0.99
Treatment (Base = *not* *participating in one of the treatments below*)						
Community treatment			1.65 ***	1.17~2.33	1.28	0.81~2.02
Institutional treatment			1.03	0.72~1.46	1.30	0.82~2.07
Reentry treatment			0.83	0.57~1.21	0.79	0.49~1.30
Employment treatment			2.07 ***	1.48~2.90	2.01 **	1.33~3.03
No confirmative experience of stigma (Base = *having confirmative experiences of stigma*)			0.81 *	0.67~0.99	0.95	0.71~1.27
No perceived stigma(Base = *having perceived stigma*)			0.99 ***	0.82~1.21	0.97	0.41~2.26
Moderation terms						
Community treatment × No perceived stigma					1.93 *	0.96~3.88
Institutional treatment × No perceived stigma					0.57	0.27~1.17
Reentry treatment × No perceived stigma					1.02	0.47~2.22
Employment treatment × No perceived stigma					1.08	0.54~2.19
No confirmative experience of stigma × No perceived stigma					0.74	0.49~1.10
Goodness-of-fit	$\chi^{2}$		$\chi^{2}$		$\chi^{2}$	
Wald test (*df*)	1036.14 *** (13)		1080.91 *** (19)		1087.97 *** (24)	
Likelihood ratio (*df*)			44.77 *** (6)		7.06 (5)	

Note. *** *p <* 0.00; ** *p <* 0.01; * *p <* 0.05. C.I. = the confidence interval. Likelihood ratio tests were conducted for logistic regression without robust standard error.

## Data Availability

The data presented in this study are available upon request from the corresponding author.
